# The Effect of Prognostic Nutritional Index in Postoperative Infection Following Lobectomy in Non-Small Cell Lung Cancer Patients

**DOI:** 10.7759/cureus.37611

**Published:** 2023-04-15

**Authors:** Seray Hazer, Selim Şakir Erkmen Gülhan, Necati Solak, Derya Yenibertiz, Mahmut Sami Akıllı, Ebru Sayilir Guven, Pınar Bıçakçıoğlu

**Affiliations:** 1 Department of Thoracic Surgery, Ankara Atatürk Sanatoryum Training and Research Hospital, Ankara, TUR; 2 Department of Thoracic Surgery, Dr. Nafiz Korez Sincan State Hospital, Ankara, TUR; 3 Department of Pulmonary Medicine, Ankara Atatürk Sanatoryum Training and Research Hospital, Ankara, TUR; 4 Emergency Department, Ankara Atatürk Sanatoryum Training and Research Hospital, Ankara, TUR

**Keywords:** lung cancer, postoperative infection, lobectomy, malnutrition, prognostic nutritional index (pni)

## Abstract

Background

The prognostic nutritional index (PNI) is a valuable marker for evaluating the nutritional status associated with postoperative complications and the prognosis of patients with cancer. However, the role and clinical value of PNI in infection after lung cancer surgery remains unclear. This study examined the association between PNI and infection after lobectomy for lung cancer, focusing on the predictive value of PNI.

Methods

We conducted this retrospective cohort study on 139 patients with non-small cell lung cancer (NSCLC) who underwent surgery between September 2013 and December 2018. Two groups were composed according to their PNI values (≥ 50 or <50 ), and the relationship was assessed with infection after lobectomy.

Results

Only PNI values, atelectasis, and prolonged air leaks were significantly associated with the development of infection. The median preoperative PNI was 52.97±5.69. Postoperative infection was seen in patients (15.5%) with PNI≥ 50 and 38.1% in patients with PNI <50. The mean PNI in patients with postoperative infection, empyema, and prolonged air leakage was lower than in patients without these conditions.

Conclusions

Malnutrition is commonly seen in patients with malignancy. The overall malnutrition rate is 45% in lung cancer patients. Patients with metastatic diseases are malnourished in a 73% ratio compared to 5% for localized diseases. Furthermore, malnutrition increases the tendency of postoperative infection and reduces wound healing. We aim to determine whether PNI can be a predictive index marker for postoperative infection in patients with NSCLC who underwent lobectomy. Postoperative infection was seen in 15.5% of patients with PNI>50 and 38.1% in patients with PNI <50.

## Introduction

This article was previously presented as an oral presentation at the 2022 Turkish Thoracic Society Annual Congress held in Antalya between May 24, 2022 and May 28, 2022. Lung cancer is a leading cause of cancer-related deaths worldwide, and about 85% of them are non-small cell lung cancer (NSCLC) [1]. Smoking status, carcinoembryonic antigen (CEA) levels, histological features, molecular markers, and staging are used to evaluate the prognosis [2]. However, a different prognosis can be observed even in patients with similar indicators, and patients may respond differently to the same treatment regimens [3]. Malnutrition is a common condition in patients with malignancy. It is reported that in lung cancer, up to 50% of patients have a risk of weight loss and malnutrition [4]. The overall malnutrition rate is 45% in lung cancer patients, and those with metastatic disease were malnourished in a 73% ratio compared to 5% for localized disease [5]. Lymphocyte count has been shown to be a prognostic factor in patients with pancreas, breast, and node-negative NSCLC. High serum albumin level, frequently used to evaluate the nutritional status of patients, is used as an indicator of good prognosis in many studies [6]. Malnutrition is associated with higher postoperative infection and in delaying wound healing. Immunosuppression is caused by dysfunction of neutrophils, macrophages, and lymphocytes, which reduces bacterial opsonization, complement activation, and production [7]. Malnutrition can cause many morbidities, significantly impaired wound healing, and bronchopleural fistula. In addition, it is an independent risk factor for length of hospital stay and costs. The most recent effective treatment for NSCLC is surgical resection. Although similar surgical techniques and wound care procedures have been applied in similar patients, infection occurs in only a few cases. There is limited data that reports the effect of the prognostic nutritional index (PNI) on lung cancer patients who underwent surgery. Most of the studies depend on surveys of NSCLC patients or gastrointestinal surgery. This study evaluated whether PNI is predictive in developing a postoperative infection after lobectomy in early-stage NSCLC. Revealing the existence of a post-lobectomy infection with PNI may help reduce postoperative infection treatment costs, hospital stays, and other related complications by using preoperative nutritional support.

## Materials and methods

We conducted this retrospective study on a total of 170 patients with NSCLC who underwent surgery at the Ankara Atatürk Sanatoryum Chest Diseases and Thoracic Surgery Training and Research Hospital in a single Thoracic Surgery Clinic between September 2013 and December 2018. Of these, 31 patients were excluded from the study. 

All the 139 patients included in the study met the following criteria: operable NSCLC; patients aged between 30 and 75 years; underwent only a single lobe resection (lobectomy) with lymph node dissection; and a PNI evaluation was obtained before surgery.

The histopathological diagnosis of the tumors was based on the WHO criteria, and the TNM stage was evaluated according to the criteria established in 2017, the 8th edition [8]. 

The exclusion criteria were: patients who were preoperative, microbiological culture positively; patients who underwent pneumonectomy, bi-lobectomy, and segmentectomy; patients who had neoadjuvant chemo-radiotherapy; and patients with inoperable and non-resectable NSCLC.

Other risk factors, such as smoking, age, surgical techniques, histological types, stage of the tumor, and BMI, were evaluated to assess for any correlation with the developed infection. Therefore the data of age, gender, histological type of NSCLC, stage, total leukocyte counts (100/L), C-reactive protein (CRP) (mg/dl), BMI (weight/height square), smoking status, surgical techniques, postoperative stay, and the other complications, were evaluated from our hospital's database. The first-generation cephalosporin was given to all the participants preoperatively.

The PNI was calculated using data from the complete blood count (CBC) routinely performed before surgery. The calculation of PNI is 10 x serum albumin level (g / dl) + 0.005 x total lymphocyte count (cells / mm3). Patients with PNI results ≥50 or <50 were categorized as either normal or low PNI groups, as established previously. In the postoperative first 10 days, sputum, aspiration materials during bronchoscopy or endotracheal intubation, pleural effusion, and wound microbiological culture-positive patients with/without antibiotic use were accepted in the patient group with infection. The study is a retrospective cohort study. A Chi-square test was used. For the sample size calculation, an alpha level of 0.05 and a beta level of 0.2 was used [9]. Assuming the OR for low PNI would be 5.58 regarding infection [10], the required total sample size was calculated as 69. This study was approved by the Keçiören Research and Training Hospital Ethics Committee (Number 2095: approved on March 2020). Informed consent was obtained from patients for this retrospective analysis of the database.

Statistical analysis

All the statistical analyses were performed using the SPSS statistical package version 22.0 (IBM Corp., Chicago, IL, USA). The descriptive statistics were reported as the number of units (n), percent (%), mean ± standard deviation (x' ± ss), and median (IQR). Pearson's chi-square and Fisher's exact test were used to evaluate categorical variables. The normal distribution of the data of numerical variables was evaluated with the Shapiro-Wilk normality test and Q-Q charts. In comparing the groups, the independent sample t-test or one-way ANOVA was used for the variables showing normal distribution. The Mann-Whitney U or Kruskal-Wallis test was used for the variables that did not fit the normal distribution. The infection detection performance of the PNI value was evaluated by plotting receiving operative characteristics (ROC) curves. Threshold values ​​were determined using the Youden index. Based on the threshold values ​​obtained, sensitivity, specificity, positive predictive, and negative predictive values ​​were calculated. Categorical data were examined using the χ2-test. Chi-square analysis was used to investigate the relationship between PNI and postoperative infection. Also, multivariate analysis (regression analysis) was used to clarify the other exposures' effect on the postoperative infection. The p<0.05 value was accepted as statistically significant.

## Results

A total of 170 patients who underwent surgery for NSCLC were retrospectively analyzed, and 139 patients were included in the study (Table 1). The mean age of the patients was 62 (56-67) years, the female-to-male ratio was 0.17 (female:male=20:119), and the median BMI was 25.8 (23.1-29.1). A total of 89.2% of the patients were active smokers, and the median pack-year smoking was 40 (range 30-50) (Table 1).

**Table 1:** Preoperative and postoperative parameters of the patients. CRP: C-reactive protein; PNI: Prognostic nutritional index.

Patients	Mean
PNI (Preoperative)	52.97±5.69
Days with drain (Postoperative)	8.00 (6.00-11.00)
Age	62.00 (56.00-67.00)
Weight (kg)	73.50 (65.00-83.25)
Height (m)	1.70 (1.65-1.74)
BMI	25.77 (23.14-29.14)
Smoking (p/y)	40.00 (30.00-50.00)
WBC (Preoperative)	7.86 (6.35-9.10)
Lymphocyte (Preoperative)	2.14 (1.75-2.60)
Albumin (Preoperative)	4.20 (4.00-4.42)
CRP (Preoperative)	0.66 (0.18-1.50)

The most common complaints were dyspnea (28.1%), cough (24.5%), chest pain (10.1%), hemoptysis (10.1%), and 27.2% of patients were asymptomatic (Table 2). The median preoperative laboratory findings were the following: WBC was 7.86 (6.35-9.10), lymphocyte was 2.14 (1.75-2.60), albumin was 4.20 g/dl (4.00-4.42), and CRP was 0.66 mg/dl (0.18-1.50) (Table 1).

**Table 2:** The most frequent complaints of the patients in admission.

Symptoms	
Dyspnea	39 (28.1%)
Cough	34 (24.5%)
Chest pain	14 (10.1%)
Hemoptysis	14 (10.1%)
Back pain	12 (8.6%)

The median preoperative PNI was 52.97±5.69 (Table 1). The lower PNI group (patients with a PNI value less than 50) included 42 patients (30.22%), and the higher PNI group (PNI more than 50) included 97 patients (69.78%). The PNI was found to be significantly higher in females than males (56.45±5.09 and 52.39±5.59, p-value = 0.003, respectively).

Eight of the patients had previously been operated on for another malignancy. The median PNI of these patients was 49.41±4.08, and the PNI values were found to be significantly lower than those without a history of previous malignancy (median PNI: 53.19±5.71) (p = 0.003). A total of 69.1% of patients underwent an upper lobectomy (n=96), 30.2% of patients underwent a lower lobectomy (n=42), and 0.7% of patients underwent a middle lobectomy (n=1). The lobectomies with lymph node dissection were performed in 56.1% of patients via a right thoracotomy, 38.13% underwent a left thoracotomy, 3.6% via right video-assisted thoracic surgery (VATS), and 2.16% via left VATS. The histopathological diagnoses were adenocarcinoma (51%, n=71), squamous cell carcinoma (39.7%, n= 55), adenosquamous cell carcinoma (5.7%, n=8), pleomorphic carcinoma (2.9%, n=4), and large cell carcinoma (0.7%, n=1), respectively. A total of 51.8% of the patients demonstrated stage I disease (n = 72), 30.9% of the patients demonstrated stage II disease (n = 43), and 17.3% of the patients demonstrated stage III disease (n = 24) (Table 3). Postoperative median hospital stay was eight days (6-11 days).

**Table 3:** The distribution of TNM classification of the patients with NSCLC. TNM: Tumor, node, and distant metastasis; NSCLC: Non-small cell lung cancer.

TNM	
T1aN0M0	5 (3.60%)
T1bN0M0	15 (10.79%)
T1bN2M0	2 (1.44%)
T1cN0M0	13 (9.35%)
T1cN1M0	1 (0.72%)
T1cN2M0	3 (2.16%)
T2aN0M0	40 (28.78%)
T2aN1M0	3 (2.16%)
T2aN2M0	6 (4.32%)
T2BN0M0	16 (11.51%)
T2bN1M0	2 (1.44%)
T2bN2M0	1 (0.72%)
T3N0M0	20 (14.39%)
T3N1M0	4 (2.88%)
T3N2M0	3 (2.16%)
T4N0M0	4 (2.88%)
T4N2M0	1 (0.72%)
Stage	
1A1	5 (3.60%)
1A2	15 (10.79%%)
1A3	13 (9.35%)
1B	39 (28.06%)
2A	16 (11.51%)
2B	27 (19.42%)
3A	20 (14.39%)
3B	4 (2.88%)

Secretion retention and minimal atelectasis were developed in 43.2% of patients during the postoperative follow-up. They were treated with flexible fiberoptic bronchoscopy. The drain removal was delayed in 9.4% of patients (n=13) due to persistent air leakage. Infection developed in 22.30% of patients (n=31) postoperatively, while 18 developed pneumonia, and empyema occurred in 13 patients (9.35%). Conditions that may be predisposed for infection include smoking, histopathological diagnosis, cancer stage, previous malignancy history, surgical technique (VATS/thoracotomy, which lobe was removed), prolonged air leakage, and atelectasis were evaluated individually (Table 3). Only atelectasis, prolonged air leakage, and PNI values were significantly related to infection. Infection developed in 35% of the patients with postoperative atelectasis (p = 0.002), with a frequency of 18.2% in patients without persistent air leaks (PAL) and 61.5% in patients with PAL in the postoperative period (p = 0.002). Postoperative infection developed in 22.3% of the patients (n=31). While the infection rate was 38.1% in patients with PNI<50, it was 15% in patients with PNI ≥50, revealing a statistically significant difference (p = 0.003). While the mean PNI was 50.38±5.09 in patients with infection, it was 53.72±5.65 in patients who did not develop infection (p = 0.007). The mean PNI in patients with empyema was 50.56±4.24, while it was 53.22±5.77 in patients who did not develop an empyema (p = 0.054). The mean PNI was 49.17±4.64 in patients with PAL; however, it was 53.36±5.65 in patients without PAL (p = 0.008).

## Discussion

Lung cancer is one of the most common cancers in both genders and the leading cause of worldwide cancer-related deaths [11]. NSCLC accounts for approximately 85% of lung cancers, with the remainder mostly being small cell lung cancer (SCLC). Most of the patients initially present in an advanced stage, and despite the use of targeted therapy, immunotherapy, and advanced surgical procedures in recent years, the results are still not very satisfactory, with most patients dying due to metastatic disease. Despite significant advances in early diagnosis and targeted therapy, the prognosis for lung cancer remains poor, with a five-year survival rate of less than 17% [12]. Smoking, carcinoembryonic antigen (CEA) levels, histological features, molecular markers, and developed staging methods are used to evaluate the prognosis [2]. However, even in patients with similar indicators, the prognosis is different. Patients may respond differently to the same treatment regimens. [3]. Tumor progression causes a systemic inflammatory response, leading to an impaired nutritional status. As a biomarker, PNI might reflect the potential cancer-related systemic inflammatory state and tumor aggressiveness. The PNI, whose measurement is based on the serum albumin level in the peripheral blood and the total lymphocyte count, is a widely related potential nutritional and immunological index marker. PNI is an independent indicator of postoperative infection in lung cancer. Initially, PNI was used to aid in assessing operative risk and evaluating perioperative nutrition and immunological conditions. Increasing evidence has revealed that preoperative nutrition and immunological status not only affect short-term postoperative complications but are also important in evaluating long-term outcomes of cancer patients [13-15]. Malnutrition is common in patients with malignancy. It is reported that up to 50% of patients with lung cancer have weight loss and malnutrition. Many different markers have been used to evaluate the nutritional status of these patients, and PNI is an effective, calculated marker determined by albumin and lymphocyte serum values [11]. It has been reported that patients with NSCLC included in the nutrition program experience fewer treatment complications. The European Lung Cancer Working Party (ELCWP) and the Japan Multinational Trial Organization (JMTO) reported that increased neutrophil rates indicate a poor prognosis in NSCLC [6]. Lymphocyte counts have been shown to be a prognostic factor in pancreatic, breast, and N0 NSCLC patients. A high serum albumin level, frequently used to evaluate a patient's nutritional status, has also been used as a good prognostic indicator in many studies [6]. Malnutrition increases the tendency for postoperative infection and reduces wound healing. Dysfunction of neutrophils, macrophages, and lymphocytes can reduce bacterial opsonization and complement activation, potentially causing immunosuppression [7]. Cancer cachexia, especially pre-cachexia, is not yet distinguished from malnutrition because of the overlap of clinical appearance [16]. Recent studies showed that PNI is a valuable biomarker to predict postoperative complications and survival in patients with completely resected NSCLC. Low PNI values have been reported as a predictor of both postoperative complications and poor prognosis in various types of malignancies. Further, low PNI correlated significantly with older age, lower BMI, larger tumor size, and elevated CRP levels [2]. In our study, the mean PNI values were significantly lower in male patients and patients with a former malignancy. The present cross-sectional study aimed to assess the association between PNI and postoperative infection of NSCLC after lobectomy. We found that PNI was an independent indicator of postoperative infection for NSCLC. In addition, PNI was significantly related to clinical characteristics, including gender and history of previous malignancy. Postoperative infection developed in 31 patients (22.3%). While the rate of infection development was 38.1% in patients with PNI<50, it was only 15% in patients with PNI>50, which was statistically significant (p = 0.003). In our study, the mean PNI was lower in patients with infection (especially in empyemas) and a PAL than in patients without the same complications. Similarly, Okada S et al. revealed in their series of 248 patients that a low PNI value was predictive for postoperative complications, PAL, and pneumonia. A five-year survival analysis study assessing nutritional status showed a significant association between a low BMI and OS. The incidence of recurrence-related death was significantly higher in the low PNI group (23.9%) than in the high PNI group (10.9%; p = 0.019) [2].

Low PNI is reported to be significantly related to postoperative complications in patients with gastric cancer and pancreatic fistula occurrence in patients with pancreatic cancer [12, 16]. Our current study of NSCLC patients identified a significant correlation between low PNI and postoperative complications. Our study also found that patients with a PNI <50 showed a significantly increased risk of postoperative infection and PAL (p = 0.003 and p = 0.001, respectively) and an increased incidence of empyema. However, that was not statistically significant (p = 0.063). Malnutrition is reportedly related to postoperative complications after lung surgery [2], and single nutritional factors were not predictive enough of broad postoperative outcomes. The PNI seems to be the most reliable factor for sensitively reflecting nutritional status and could help assess the potential incidence of pulmonary complications. Generally, surgical trauma is common, and results in surgical stress, and the inflammatory response could reduce albumin levels. Hypo-albuminemia is considered a risk factor for postoperative complications [17]. If the patient is not able to maintain adequate nutrition, oral supplementation, enteral (tube) feeding, or parenteral (IV) feeding may be used for nutritional intervention. Enteral support is recommended over parenteral support because of its relative simplicity, safety, reduced complications, and lower cost [15]. Preoperative parenteral feeding increases the risk of infectious complications. A study evaluated the nutritional risk of 1085 patients defined by the Nutritional Risk Screening Tool 2002 (NRS-2002) and the effect of preoperative nutrition support in 512 patients undergoing abdominal surgery [18]. Of the 120 patients with an NRS score ≥5, the complication rate was significantly lower in the preoperative nutritional group compared with the control group (25.6 versus 50.6%). The length of hospital stay was significantly shorter in the preoperative nutritional group than in the control group (13.7 versus 17.9±11.3 days). No significant differences were seen for lesser NRS scores. Some limitations must be considered in analyzing these results. The study was a retrospective observational analysis, and some residual confounding factors remain. It was a single-center study, and our local experience might influence the outcome of perioperative management strategies. Therefore, a large, multicenter, randomized controlled trial should be performed to assess and confirm our study results. A prospective study may be developed to determine the effect of providing nutritional support before lobectomy for malignancy patients with a low PNI value.

## Conclusions

PNI is associated with an increased risk of infection after lobectomy in NSCLC patients and increases the risk of other postoperative complications. Low PNI values negatively affect the patient's recovery process.
